# Elevation and plant species identity jointly shape a diverse arbuscular mycorrhizal fungal community in the High Arctic

**DOI:** 10.1111/nph.18342

**Published:** 2022-07-15

**Authors:** Pil U. Rasmussen, Nerea Abrego, Tomas Roslin, Maarja Öpik, Siim‐Kaarel Sepp, F. Guillaume Blanchet, Tea Huotari, Luisa W. Hugerth, Ayco J. M. Tack

**Affiliations:** ^1^ Department of Ecology, Environment and Plant Sciences Stockholm University SE‐106 91 Stockholm Sweden; ^2^ The National Research Centre for the Working Environment 105 Lersø Parkallé DK‐2100 Copenhagen Denmark; ^3^ Department of Agricultural Sciences University of Helsinki PO Box 27, (Latokartanonkaari 5) Helsinki FI‐00014 Finland; ^4^ Department of Ecology Swedish University of Agricultural Sciences Box 7044 Uppsala SE‐750 07 Sweden; ^5^ Department of Botany University of Tartu 40 Lai Street Tartu 51005 Estonia; ^6^ Département de Biologie, Faculté des Sciences Université de Sherbrooke 2500 Boulevard Université Sherbrooke QC J1K 2R1 Canada; ^7^ Département de Mathématiques, Faculté des Sciences Université de Sherbrooke 2500 Boulevard Université Sherbrooke QC J1K 2R1 Canada; ^8^ Département des Sciences de la Santé Communautaire, Faculté de Médecine et des Sciences de la Santé Université de Sherbrooke 3001 12^e^ Avenue Nord Sherbrooke QC J1H 5N4 Canada; ^9^ Department of Molecular, Tumor and Cell Biology, Science for Life Laboratory, Center for Translational Microbiome Research Karolinska Institutet SE‐171 65 Solna Sweden

**Keywords:** abiotic and biotic environment, altitudinal gradient, arbuscular mycorrhizal (AM) fungi, climate, elevational gradient, High Arctic

## Abstract

- Knowledge about the distribution and local diversity patterns of arbuscular mycorrhizal (AM) fungi are limited for extreme environments such as the Arctic, where most studies have focused on spore morphology or root colonization. We here studied the joint effects of plant species identity and elevation on AM fungal distribution and diversity.
- We sampled roots of 19 plant species in 18 locations in Northeast Greenland, using next generation sequencing to identify AM fungi. We studied the joint effect of plant species, elevation and selected abiotic conditions on AM fungal presence, richness and composition.
- We identified 29 AM fungal virtual taxa (VT), of which six represent putatively new VT. Arbuscular mycorrhizal fungal presence increased with elevation, and as vegetation cover and the active soil layer decreased. Arbuscular mycorrhizal fungal composition was shaped jointly by elevation and plant species identity.
- We demonstrate that the Arctic harbours a relatively species‐rich and nonrandomly distributed diversity of AM fungi. Given the high diversity and general lack of knowledge exposed herein, we encourage further research into the diversity, drivers and functional role of AM fungi in the Arctic. Such insight is urgently needed for an area with some of the globally highest rates of climate change.

## Introduction

Arbuscular mycorrhizal (AM) fungi are important root symbionts found in the majority of terrestrial plant roots (van der Heijden *et al*., 2015). Arbuscular mycorrhizal fungi can have significant impacts on plant fitness (Klironomos *et al*., 2000), plant community composition (Hartnett & Wilson, 1999; van der Heijden *et al*., 2008) and ecosystem functioning (van der Heijden *et al*., 2015) – although in other cases, AM fungi have been found to colonize plant roots with few benefits for the plant (Cosme *et al*., 2018; Wang *et al*., 2021, 2022). Since the advent of modern sequencing techniques, the global diversity patterns of AM fungi have become increasingly well understood (Kivlin *et al*., 2011; Davison *et al*., 2015, 2018). Although many AM fungal species are distributed globally (Davison *et al*., 2015), others are confined to particular habitats or geographical regions (Veresoglou *et al*., 2013; Davison *et al*., 2016) and may show remarkable niche differentiation, in particular in relation to temperature and soil pH (Davison *et al*., 2021). However, for some major land areas, including the Arctic, AM fungal diversity and its drivers remain poorly understood (Öpik *et al*., 2013; Pärtel *et al*., 2017), even though AM fungal communities in the Arctic are distinct from those in other areas (Vasar *et al*., 2022).

As the Arctic regions are crucial for the storage of large portions of the Earth's carbon (C) stocks (Mack *et al*., 2004), it is important to understand the potential drivers of these stocks. Mycorrhizal fungi, including AM fungi, may contribute to C cycling and storage via impacts on plant photosynthetic rates, use of photosynthates, and C storage in their biomass (Read & Perez‐Moreno, 2003; Godbold *et al*., 2006; Soudzilovskaia *et al*., 2015a,b; Deckmyn *et al*., 2020). Arctic regions are currently experiencing the globally highest rates of climate change (IPCC, 2014), so we urgently need to understand the general diversity and role of AM fungi in cold climates, and the environmental drivers of local AM fungal communities.

Compared to temperate environments, few studies exist on the regional species pool of AM fungi in Arctic environments, the relative abundance of taxa and the structuring of AM fungal communities along environmental gradients. The existing studies from the Arctic typically have relied on either spore morphological identification from soil samples (with some uncertainty about host plant identity), or root colonization quantification (bringing little information about AM fungal species diversity) (although see Appoloni *et al*., 2008; Öpik *et al*., 2013; Davison *et al*., 2018).

In a study along a latitudinal gradient in the Canadian Arctic, Olsson *et al*. (2004) found high AM fungal root colonization at the southernmost sites, but little to no colonization at the northernmost sites, despite the presence of putative AM plants. The most northern sites did, however, sustain a higher degree of non‐mycorrhizal plants. A higher abundance of non‐mycorrhizal and facultative mycorrhizal plants in harsh environments is a common pattern (Bueno *et al*., 2017). For the Arctic, several explanations have been proposed for the low prevalence of AM plants, as reviewed in Kytöviita (2005). One hypothesis attributes this pattern to history, because the Arctic ecosystem has evolved relatively recently and been deglaciated for only 3000–8000 yr. Another notion is that mycorrhizal associations with higher degradative abilities, such as ericoid mycorrhiza or ectomycorrhiza, will provide a larger benefit to plants, and therefore be more prevalent. Finally, Kytöviita (2005) proposes that the low prevalence of AM fungi might be a consequence of poor adaptation by AM fungi to nutrient uptake in cold environments. From the perspective of an Arctic plant, the costs for sustaining an AM fungal partner may then outweigh the benefits. With regards to AM fungal colonization of roots, several studies from the Arctic nonetheless have found colonization levels ranging from 11–36% root length colonized (Allen *et al*., 2006), through 27–51% root length colonized (Ormsby *et al*., 2007), to 37–85% root length colonized (Olsson *et al*., 2004). Newsham *et al*. (2017) studied 102 plants from 11 plant species, and found structures resembling AM fungi in 41 of the plant individuals.

To date, few studies have investigated the diversity of AM fungi in the Arctic. As these studies are based mostly on soil samples taken from a mixed rhizosphere, there is some uncertainty about the link between AM fungal diversity and host plant identity. For example, Varga *et al*. (2015) used spore morphotyping from soil samples to find 18 spore morphospecies, and Greipsson *et al*. (2002) used trap‐culturing and spore morphotyping to discover 11 morphospecies. Some DNA‐based root and soil AM fungal data exist from Iceland, Svalbard and the Scandinavian Arctic (Appoloni *et al*., 2008; Öpik *et al*., 2013; Davison *et al*., 2015, 2018; García de León *et al*., 2018). Here, the species concept most frequently adopted is that of Virtual Taxa (VT; Öpik *et al*., 2010), for which most studies have shown moderate diversity of approximately 10–20 VT per area. For example, in Iceland, Norrbotten (Sweden), and Lapland (Finland), authors found species within the genera Glomeraceae, but also a few Acaulosporaceae, Claroideoglomeraceae and Diversisporaceae (Appoloni *et al*., 2008; Öpik *et al*., 2013; Davison *et al*., 2018; García de León *et al*., 2018). Of those identified to VT, the Maarj*AM* database showed that 9, 19 and 22 AM fungal VT have been found in these areas, respectively.

Overall, although studies suggest that the presence of AM fungal symbiosis is low in the Arctic at the level of both plant species (Allen *et al*., 2006; Newsham *et al*., 2017) and individuals (Newsham *et al*., 2017), cases of high root colonization by AM fungal structures have still been reported (Olsson *et al*., 2004; Ormsby *et al*., 2007), as have several species of AM fungi (Greipsson *et al*., 2002; Öpik *et al*., 2013). It thus appears that there is still much to learn about AM fungal diversity in the Arctic and how it relates to plant species identity.

A particular knowledge gap relates to the impact and relative importance of plant species identity in structuring AM fungal communities, and how the influence of plant species identity varies along environmental gradients (Helgason & Fitter, 2009; Vályi *et al*., 2016). Elevational gradients are convenient to address this topic, because they show strong variation in the abiotic and biotic environment (e.g. in temperature, resource availability and vegetation structure) at fine spatial scales (Körner, 2007). Simultaneously, AM fungal richness, root colonization and spore density have been found to decrease with increasing elevation (Gai *et al*., 2012). Even though many AM fungal species are able to colonize a large range of plant species, there is evidence that plant identity can leave a detectable imprint on AM fungal community composition (Vandenkoornhuyse *et al*., 2003; Hausmann & Hawkes, 2009; Sepp *et al*., 2019; Davison *et al*., 2020). Additionally, studies have found that elevational gradients may add a further signature to plant–AM fungal associations: Li *et al*. (2014) reported that AM fungal communities in two plant species were more similar at intermediate elevations than at low or high elevations, respectively. Whether Arctic AM fungal communities respond to such environmental gradients remains to be resolved.

In order to study how variation in environmental conditions and plant species identity influence the distribution of AM fungi within the High Arctic, we used an elevational gradient located in the Zackenberg valley, Northeast Greenland. We identified AM fungi by amplicon‐sequencing the roots of 19 Arctic plant species, sampled at 18 locations along the elevational gradient. At each sampling location, we characterized the abiotic and biotic environment. We targeted the following questions:

(**1**) What is the species richness and composition of AM fungal communities in the High Arctic?
(**2**) What are the relative and joint impacts of elevation and plant species in explaining the presence, richness, composition and network structure of AM fungal communities?
(**3**) How do the abiotic and biotic factors varying along elevation influence AM fungal occurrence, richness and community composition?

Based on global patterns in the structuring of mutualistic associations, we expected Arctic AM fungal species to be generalists (Schleuning *et al*., 2012), able to live in a broad range of habitats, to be globally widespread (Orme *et al*., 2006) and to lack unique adaptation to the Arctic or local environment (e.g. Öpik *et al*., 2006; Davison *et al*., 2015). Based on records from the Maarj*AM* database (maarjam.ut.ee), we expected to find the species richness to be in the range of 5–25 VT.

## Materials and Methods

### Study system

The Zackenberg valley, Northeast Greenland (lat. 74°30′N, long. 21°00′W; Supporting Information Fig. S1) is part of the High Arctic climate zone, characterized by mean monthly temperatures ranging from −20°C to +7°C and by an annual precipitation of 260 mm. The low Arctic vegetation of the area is relatively rich and diverse (Bay, 1998), with the most typical plants being arctic willow (*Salix arctica*), arctic bell‐heather (*Cassiope tetragona*) and mountain avens (*Dryas*). We note that most individuals of *Dryas* in northeastern Greenland are interspecific hybrids (*Dryas octopetala* × *integrifolia*) (Philipp & Siegismund, 2003).

### Study design

Samples were collected in July 2015 in 18 locations on the western slope of the Aucella Mountain, ranging from 33 to 479 m above sea level (Fig. S1). The sampling locations were randomly located along the elevational gradient, with a distance between sites ranging from 373 m to 6.4 km, with an average of 2.7 km (Fig. S1). We followed a two‐step sampling protocol with the aim to (1) characterize the AM fungal community associated with the broader plant community (which often includes plant species present at only one or a few sampling locations), and (2) assess the impact of elevation on AM fungal richness and community composition in a set of key plant species present across the elevational gradient (Bay, 1998). We note that the plant species examined in this study were sampled as part of a previous project with distinct aims (Abrego *et al*., 2020a,b). In that project, the aim was to study the effects of elevation and environment on a broad group of root‐associated fungi (including other groups of mycorrhizal fungi). However, because the primers used provided low detection and resolution of AM fungi, we took advantage of the same, unique collection of DNA samples to gain a deeper knowledge on the occurrence and diversity of AM fungi.

At each transect, we sampled the most common plant species, including plant species which are non‐mycorrhizal. This may have led to a lower detected AM fungal diversity compared to if only AM plants had been sampled. The set of key plant species sampled consisted of alpine bistort (*Bistorta vivipara*), white arctic bell‐heather (*Cassiope tetragona*), mountain avens (*Dryas octopetala × integrifolia)* (Elkington, 1965; Philipp & Siegismund, 2003), arctic willow (*Salix arctica*), purple saxifrage (*Saxifraga oppositifolia*) and moss campion (*Silene acaulis*).

Within each sampling location, we first sampled the roots of five individuals of each of the six key species found along a 50‐m transect along a given elevation, with the distance among samples from conspecific individuals being ≥ 1 m apart. From each plant, the whole root system was uprooted, and the fine roots (< 2 mm) collected. Second, we collected roots of one individual plant from each of five of the other most common plant species along the 50‐m transect. We thereby sampled the majority of the most common plants at each transect (Table S1). Some of the nonkey plant species were sampled only at a single location, whereas others were sampled from several locations (Table S1).

Roots were cleaned of soil particles by hand (first in the field and later in the laboratory to verify the absence of soil particles under a magnifying lens), wrapped in tissue paper and dried in plastic bags containing moisture‐indicating silica gel. During field sampling, at three points separated by 25 m within each sampling location, we also measured the following environmental variables directly in the field: soil pH (in soil–water suspension, using a Direct Soil Measurement pH Portable Meter; Hanna Instruments, Kungsbacka, Sweden), soil moisture (%; measured using a HydroSense Handheld Soil Moisture Sensor; Campbell Scientific, Logan, UT, USA), the depth of the active layer (cm; measuring the distance until the frozen horizon with a metal bar), distance to the nearest snow patch (m) and vegetation cover (%; visually estimating the vascular plant cover in a 1 × 1 m area; Figs S2, S3). The averages of the three replicates then were calculated to construct our environmental variables.

In order to study the AM fungal community, we took a three‐step approach. First, we used the literature to exclude those plant species (three of 25) that were *a priori* known to be ericoid mycorrhizal. We included plants where the literature indicated that the plants are nonmycorrhizal (column 2 in Table S2). Second, for the set of species remaining (*n* = 22), we tested whether DNA from samples could be amplified using primers typically used for, but not completely specific to, AM fungi (described in more detail as ‘Part 1: pilot study’ under Molecular methods in the Materials and Methods section; Table S2). Based on the amplification results, we chose a final set of plant species from which AM fungi were more thoroughly amplified and sequenced (*n* = 19; 424 samples in total; ‘Part 2: sequencing’ in the Materials and Methods section; Table S2). Four of the 19 plant species were key plant species, which are henceforth referred to as ‘focal species’.

Importantly, we used the full amount of root samples for DNA extractions. For this reason, no root material remained for microscopical investigation of AM fungal structures or root colonization levels. Thus, we were unable to verify whether and to what extent the detection of AM fungal DNA was matched by arbuscular mycorrhizal structures within roots. Given the nature of our data, and given prior demonstrations of nonmutualistic colonization of nonhost plant roots by AM fungi (e.g. Cosme *et al*., 2018; Wang *et al*., 2021, 2022), we explicitly avoid using the presence of DNA of AM fungi (Glomeromycotina) as evidence for the existence of arbuscular mycorrhizal structures or symbiotic relations between plants and AM fungi.

### Molecular methods

In order to detect and identify AM fungi efficiently, we used primers which targeted a fragment of the small subunit rRNA gene of Glomeromycotina (NS31: Simon *et al*., 1992; AML2: Lee *et al*., 2008). Abrego *et al*. (2020a,b) previously examined the whole root‐associated fungal community in the same samples. As their aim was to resolve differences in specialization along the elevational gradient between mycorrhizal (mainly ectomycorrhizal) and endophytic fungi, they used primers targeting the ITS2 region (ITS4: White *et al*., 1990; fITS7 Ihrmark *et al*., 2012) to capture most of the fungal community (Schoch *et al*., 2012). However, these primers have been shown to be inefficient at capturing AM fungi, which were expected to be present at low diversity and abundance levels at the study site (Lekberg *et al*., 2018).

The dried root samples were ground using a ball mill (Mixer Mill MM400; Retsch, Haan, Germany) and 10 mg then was used for DNA extraction using NucleoSpin Plant II kit (Machery‐Nagel, Düren, Germany). For samples < 10 mg, we used the entire sample (32 of 424 samples, mean ± SD: 7.4 ± 1.9 mg). For PCR amplification, we used the primers NS31 and AML2, which target a *c*. 560‐bp central fragment of the SSU rRNA gene in the Glomeromycotina (Simon *et al*., 1992; Lee *et al*., 2008). Note that the 18S gene region has been criticized for lacking sufficient resolution (see, e.g. Kohout *et al*., 2014, for a comparison of primers for AM fungi).

#### Part 1: pilot study

In the first part, we tested whether the plant species chosen for our pilot study amplified DNA using these primers – namely, potential AM fungal DNA (Table S2). This was done by PCR amplification, with a PCR mixture consisting of 15 μl Kapa HiFi Mastermix (Kapa Biosystems, Woburn, MA, USA), 10 μl H_2_O, 1.5 μl of each primer (5 nmol μl^−1^), and 2 μl of 4 ng μl^−1^ DNA template. PCR was conducted on the MasterCycler Pro S (Eppendorf, Hamburg, Germany). Cycling conditions were 95°C for 5 min, 98°C for 1 min, 36 cycles of 98°C for 40 s, 58°C for 40 s, and 72°C for 15 s, followed by a final elongation step of 72°C for 5 min. To test whether potential AM fungal DNA was amplified, we ran the PCR products on an 2100 Bioanalyzer (Agilent, Santa Clara, CA, USA).

#### Part 2: sequencing

Based on the results in the pilot study, we selected 19 of the 22 plant species for Illumina MiSeq sequencing. Samples from the selected plant species were PCR‐amplified in two steps following Rasmussen *et al*. (2018). In short, the first PCR reaction followed the same procedure as was previously described but with 25 cycles of 98°C instead of 36. The primer here consisted of the adaptor and primer (with the latter identified in bold face), resulting in the forward primer 5′‐TCGTCGGCAGCGTCAGATGTGTATAAGAGACAG**TTGGAGGGCAAGTCTGGTGCC**‐3′ and the reverse primer 5′‐GTCTCGTGGGCTCGGAGATGTGTATAAGAGACAG**GAACCCAAACACTTTGGTTTCC**‐3′. In the second PCR step 15 μl PCR template, 20 μl Kapa HiFi Mastermix and 2.5 μl of each primer (10 nmol μl^−1^) were used. The primers for the second PCR reaction comprised the Illumina handle, barcode and adaptor, resulting in the primers 5′‐AATGATACGGCGACCACCGAGATCTACAC‐X8‐TCGTCGGCAGCGTC and 5′‐CAAGCAGAAGACGGCATACGAGAT‐X8‐GTCTCGTGGGCTCGG‐3′, with X8 denoting unique tags of 8 bp. Reaction conditions for the second PCR were as described above, but with 11 cycles instead of 25. The final product was pooled and sent to sequencing at SciLifeLab/NGI (Solna, Sweden) on a MiSeq apparatus (Illumina Inc., San Diego, CA, USA) with 2 × 300‐bp reads.

### Bioinformatics

Each read pair was trimmed to remove primer sequences and 3′‐bases with a Phred score < 15, using cutadapt (Martin, 2011). Read pairs not containing both primers, reads with an expected error rate > 15% and any read of a length < 120 bp after trimming were discarded. Reads then were merged using Vsearch (Rognes *et al*., 2016). The total number of reads discarded because of low quality or inability to merge was 5–20%. Reads then were dereplicated and denoised using unoise3 with the minsize = 4 (Edgar, 2016). The resulting 1662 amplicon sequence variants (ASV; 18 218 806 reads; Callahan *et al*., 2017) were subjected to a Blast+ (Camacho *et al*., 2009) search with a minimum identity of 95% and 95% alignment (length of alignment/length of query) against an in‐house database, which consisted of the Maarj*AM* database (Öpik *et al*., 2010) and VT not yet in Maarj*AM*. The in‐house database contained 475 taxa represented by a total of 22 961 sequences trimmed to the region between the NS31 and AML2 primers. One hundred and nine ASV (6.5%) were identified as Glomeromycotina in this step. The remaining ASV (1553 of the 1662 ASV, 93.5%) then were blasted against GenBank with 95% identity and 90% alignment; for sequences identified to Glomeromycotina (1676 sequences belonging to 16 ASV), a bootstrapped neighbour‐joining phylogenetic analysis was run against the in‐house database type sequences and some outgroups using Mafft v.7 (Katoh *et al*., 2002) (see Methods S1; Notes S1 for further details and phylogenetic tree in Newick format). The potential new VT were assessed on the phylogenetic tree and double‐checked against the entire Maarj*AM* database. This resulted in six new VT, some consisting of more than one ASV. Arbuscular mycorrhizal fungal species accumulation curves showed that AM fungal species numbers approached an asymptote within the data range for the four focal plant species, though plant species that were sampled much less did not (Fig. S4). Sequencing data for each sample and representative sequences for each VT in this study have been deposited as a BioProject at INSDC under accession no. PRJEB40490.

### Statistical analysis

In order to describe the diversity of AM fungi at our High Arctic sampling site, we focused on the full set of 19 plant species, including 424 plant individuals. To investigate the effects of elevation, plant species identity and environmental parameters on AM fungal occurrence, richness and community composition, we focused on the subset of plants that were sampled in a balanced design across the entire elevational gradient (our so‐called ‘focal plants’, which consisted of 360 plant individuals from four plant species).

More specifically, to investigate the impact of elevation and plant species on AM fungi, we modelled the presence–absence (*n* = 360), species richness (conditional on presence, *n* = 80) and community composition (*n* = 360) of AM fungi as a function of ‘elevation’, ‘plant species’ and ‘elevation × plant species’. Here, we use elevation as a catch‐all term that characterizes variation in multiple different conditions changing in concert along the mountain slope, and thus includes the biotic and abiotic environment such as temperature, precipitation, soil nutrients, vegetation cover and diversity, soil depth and potentially more. Adding a second‐order term to elevation (representing a nonlinear pattern) did not improve the model performance (as based on ΔAIC). As such, we only included the linear term. The continuous variable ‘elevation’ was scaled to a mean of 0 and unit variance before analyses. To account for variation among sampling locations, we added ‘sampling location’ as a random effect for the presence–absence and species richness models. For the species richness and community composition models, we took the heuristic solution to include the square root of the read count to account for differences in sequencing depth among samples. Such a functional relation will accommodate the asymptotic relationship between species richness and sample size assumed in both intrapolation (rarefaction; Hurlbert, 1971; Simberloff, 1972) and extrapolation (chao estimators; Chao, 1987) across sample size.

For the presence–absence and species richness models, we used generalized linear mixed effects models (GLMM). For presence–absence data, we assumed a binomial error distribution with a logit link function; for log_10_‐transformed data on species richness we assumed a normal distribution of errors and an identity link. In models of community composition, we detected collinearity between elevation and sampling location. Therefore, to measure the confounding effect of ‘sampling location’ on elevation, we first ran a canonical redundancy analysis (RDA; Legendre & Legendre, 2012, section 11.1) on the Hellinger pre‐transformed (Legendre & Gallagher, 2001) AM fungal community data, where individual samples were aggregated at the sampling location level, using only the effect of elevation as an explanatory variable. The idea behind performing this analysis was to study the importance of sampling location for the AM community. This was meant to give us insight about the coarse factor structuring the community. As this was nonsignificant, we continued analyzing the full model in which we could now exclude sampling location.

We then defined RDA models on the nonaggregated AM community data. Before building these models, we applied a Hellinger pre‐transformation on the data. We first carried out an RDA model that included ‘plant species’, ‘elevation’ and ‘square root of the read count’ as explanatory variables to measure the importance of the main effects. Next, we included the interaction between elevation and plant species in addition of the other explanatory variables, to quantify the importance of this interaction in structuring the AM community. We then tested the marginal effects of each term of the RDA models with a permutation test (999 permutations were used to test each marginal term).

In order to test for tentative effects of abiotic and biotic variables varying along the elevation on AM fungi, we modelled the presence–absence (*n* = 360), species richness (conditional on presence, *n* = 80) and community composition (*n* = 360) of AM fungi as a function of ‘soil pH’, ‘soil moisture, ‘depth of the active soil layer’, ‘distance to nearest snow patch’ and ‘vegetation cover’. ‘Sampling location’ and ‘plant species’ were included in the presence–absence and richness models as random effects. We also included the square root of the read count to account for differences in sequencing depth among samples for the richness and community composition models. The same model types and transformations were used as described above.

We tested whether there was spatial autocorrelation in AM fungal richness and community composition (Diniz‐Filho *et al*., 2003). This was, for AM fungal richness, done by fitting the full models as described above (one for plant species identity and elevation, and the other for the environmental variables), and then examining the residuals of the model for spatial autocorrelation. We here used Moran's test implemented in the function *Moran.I* in the package ape (Paradis & Schliep, 2019). For community composition, a correlogram was used, using the function *mantel* in the vegan package. Based on these analyses we found no indications of significant spatial autocorrelation.

For the analyses, we used the packages lme4 v.1.1–21 (Bates *et al*., 2014), car v.3.0‐2 (Fox & Weisberg, 2011) and vegan v.2.5‐4 (Oksanen *et al*., 2015) in R v.3.5.2 (R Core Team, 2018). The lmerTest package (Kuznetsova *et al*., 2015) was used to test the significance of random effects, and the MuMIn package (Barton, 2019) was used to calculate the coefficient of determination (*R* ^2^) for significant fixed effects, as well as for random effects. For significant fixed effects, we calculated the marginal *R* ^2^ by running a model with the same random effect structure and a single significant fixed effect – thus, excluding other fixed effects. For determining *R* ^2^ for random effects, we calculated the conditional *R* ^2^ by fitting a model including only the random effect term. To visualize AM fungal and plant associations among elevational zones we used the package bipartite (Dormann *et al*., 2008).

## Results

**Fig. 1:**
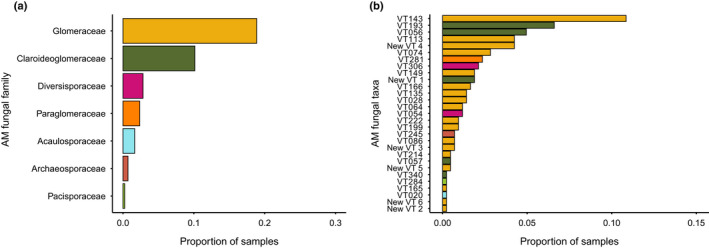
Taxonomic distribution of arbuscular mycorrhizal fungal families found in samples collected at Zackenberg, Greenland at the (a) family and (b) virtual taxa (VT) level. In (b), colours correspond to the family level to which the VT belong: dark yellow, Glomeraceae; green, Claroideoglomeraceae; pink, Diversisporaceae; bright orange, Paraglomeraceae; light blue, Acaulosporaceae; orange brown, Archaeosporaceae; light green, Pacisporaceae.

We detected 29 AM fungal VT occurring in 105 of the 424 plant individuals investigated (17 471 AM fungal reads, for positive samples on average 166 ± 709 reads (SD)). Arbuscular mycorrhizal fungi were present in 14 of the 19 plant species studied. Of the AM fungal VT, 23 were described previously as VT and six were novel AM fungal VT. Five of the novel AM fungal VT belonged to *Glomus* and one to *Claroideoglomus*. The Glomeraceae and Claroideoglomeraceae were the most diverse taxa and were present in the highest proportion of samples (Fig. 1a; Table 1). Members of Diversisporaceae, Paraglomeraceae, Acaulosporaceae, Archaeosporaceae and Pacisporaceae also were detected (Fig. 1a; Table 1). The most common VT belonged to *Glomus* (VT143 – uncultured, VT113 – related to *Rhizophagus irregularis*, and a new AM fungal taxon, ‘New VT 4’) and *Claroideoglomus* (VT193 – related to *C. claroideum‐etunicatum* group, VT56 – uncultured; Fig. 1b).

**Table 1:** Arbuscular mycorrhizal fungal families detected in the samples, along with the number of virtual taxa (VT) resolved per family, the number of reads attributed to this family, the proportion of reads and the proportion of samples testing positive for that family.

	VT	Number of reads	Proportion of reads	Proportion of samples
Acaulosporaceae	1	404	0.02	0.02
Archaeosporaceae	1	39	0.002	0.01
Claroideoglomeraceae	5	5782	0.33	0.10
Diversisporaceae	2	472	0.03	0.03
Glomeraceae	18	10 655	0.61	0.19
Pacisporaceae	1	43	0.003	< 0.001
Paraglomeraceae	1	76	0.004	0.02

**Fig. 2:**
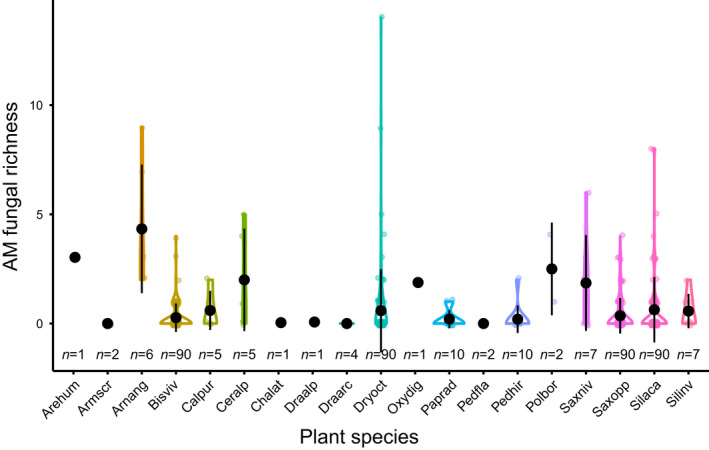
Arbuscular mycorrhizal (AM) fungal richness per sample in the roots of the studied plant species. Shown are violin plots for each plant species with mean ± SD in black. Each sample is shown as a coloured dot, with the colour corresponding to plant species. The number of samples sequenced for each plant species (*n*) can be seen below each violin plot. Arehum, *Arenaria humifusa*; Armscr, *Armeria scabra*; Arnang, *Arnica angustifolia*; Bisviv, *Bistorta vivipara*; Calpur, *Calamagrostis purpurascens*; Ceralp, *Cerastium alpinum*; Chalat, *Chamerion latifolium*; Draalp, *Draba alpina*; Draarc, *Draba arctica*; Dryoct, *Dryas octopetala x integrifolia*; Oxydig, *Oxyria digyna*; Paprad, *Papaver radicatum*; Pedfla, *Pedicularis flammea*; Pedhir, *Pedicularis hirsuta*; Polbor, *Polemonium boreale*; Saxniv, *Saxifraga nivalis*; Saxopp, *Saxifraga oppositifolia*; Silaca, *Silene acaulis*; Silinv, *Silene involucrata*.

Arbuscular mycorrhizal fungal richness was 2.3 ± 2.1 (mean ± SD) VT per plant individual (excluding plant individuals with no AM fungi; Fig. 2). The highest AM fungal richness per plant individual were found in the roots of *Arnica angustifolia* (4.3 ± 2.9), *Polemonium boreale* (2.5 ± 2.1), *Cerastium alpinum* (2.0 *±* 2.4) and *Saxifraga nivalis* (1.9 ± 2.2). Ten plant species yielded individual samples both with and without AM fungi (plant individuals without AM fungi: *B. vivipara* = 72 of 90, *Calamagrostis purpurascens* = three of five, *Cerastium alpinum* = two of five, *Dryas octopetala × integrifolia* = 71 of 90, *Papaver radicatum* = eight of 10, *Pedicularis hirsuta* = nine of 10, *S. nivalis* = three of seven, *S. oppositifolia* = 71 of 90, *Silene acaulis* = 66 of 90, *S. involucrata* = four of seven; Fig. 2). The highest recorded AM fungal richness was found in a single *D. octopetala × integrifolia* individual (14 VT).

**Fig. 3:**
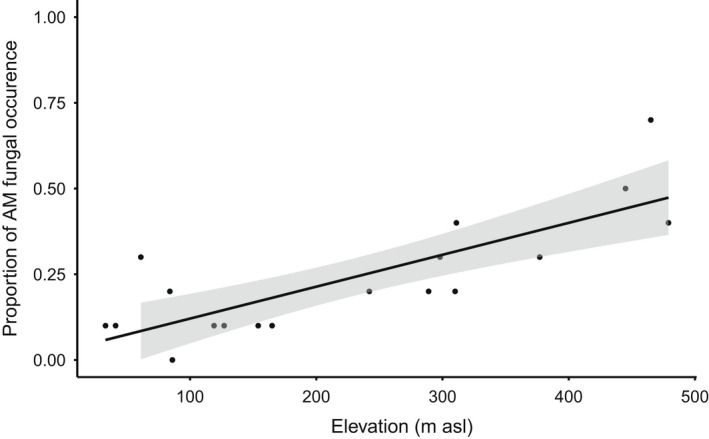
Proportion of arbuscular mycorrhizal (AM) fungi over the elevational gradient. Black circles, proportion AM fungal positive plants at each sampling location. Shown is a trendline (black) with its SE interval (grey) made with the function *geom_smooth* from R/ggplot2.

**Fig. 4:**
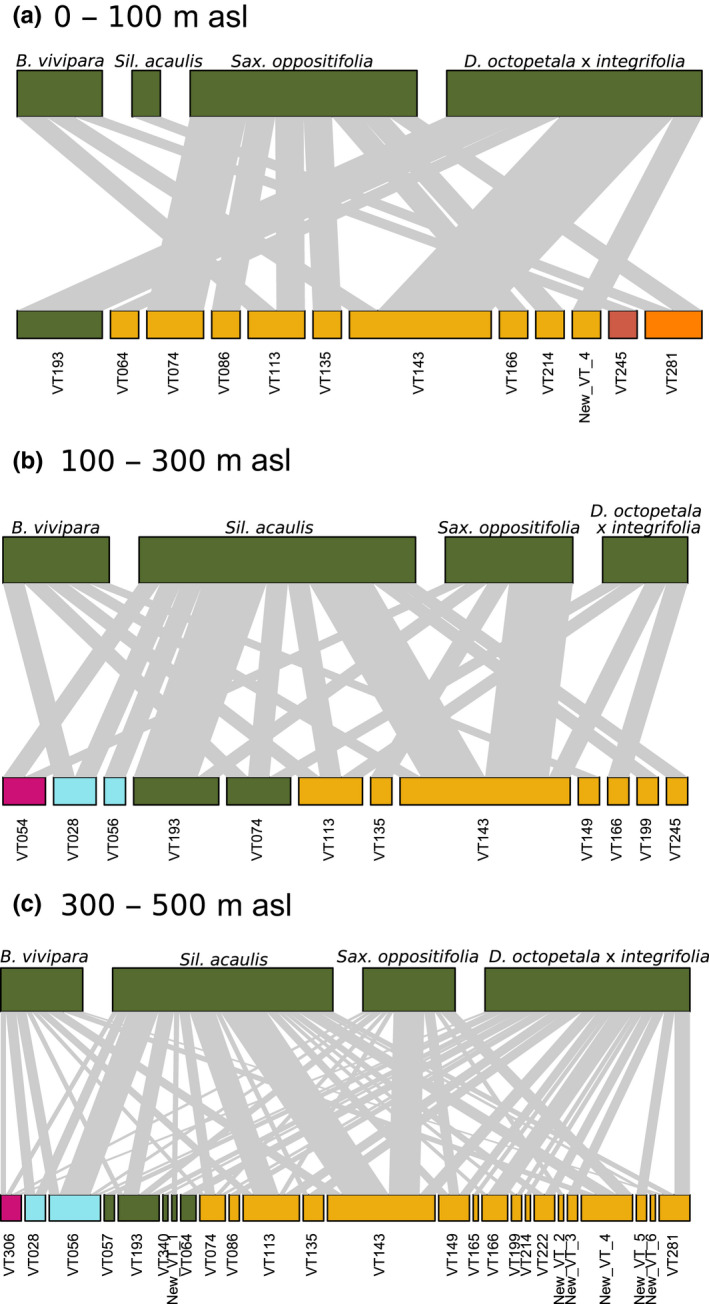
Bipartite network illustrating associations among the four best‐sampled plant species and arbuscular mycorrhizal (AM) fungal taxa found at (a) low (0–100 m above sea level), (b) mid (100–300 m asl) and (c) high (300–500 m asl) elevation in Zackenberg, Greenland. The width of the upper bars reflects the abundance of plant species, the width of the lower bars reflects the abundance of AM fungal taxa in terms of the number of samples in which they were found, and the links from plants to AM fungi show the proportion of samples from which an AM fungal taxon is found for each of the focal plant species. Bars of AM fungal taxa are coloured and ordered by relatedness at the family level, and ordered numerically within family. Colours for AM fungi correspond to Fig. 2: pink, Diversisporaceae; light blue, Acaulosporaceae ; green, Claroideoglomeraceae; dark yellow, Glomeraceae ; orange brown, Archaeosporaceae; bright orange, Paraglomeraceae.

Whether or not any AM fungi were present in the sample was best explained by elevation, with AM fungi found in more samples at higher elevations (Fig. 3; Table 2). By contrast, AM fungal richness was best explained by plant species identity (Figs 2, S5; Table 2). Arbuscular mycorrhizal fungal community composition was structured by the joint effects of elevation and plant species (Figs 4, S6; Table 2). As more AM fungal species were present at higher elevations, the number of AM fungal and plant species links increased – thus, all focal plant species were linked to the largest number of AM fungal species at the highest elevation (Fig. 4). At low elevations, most VT associated with only one plant species, whereas at higher elevation, many of the same VT associated with several focal plant species (Fig. 4). At the highest elevation, five of the six novel VT associated with a single plant species, whereas one (New VT 4) associated with three of the focal plant species (*B. vivipara, S. acaulis* and *S. oppositifolia*; Fig. 4).

**Table 2:** Impact of elevation, plant species and their interaction on the presence–absence of arbuscular mycorrhizal (AM) fungi, richness in samples positive for AM fungi, and community composition in the four focal plant species (*Bistorta vivipara, Dryas octopetala x integrifolia, Saxifraga oppositifolia, Silene acaulis*), as based on generalized linear mixed effects models (GLMM) and redundancy analysis (RDA).

	Presence–absence			Richness				Community composition		
	df	*χ* ^*2*^	*P*	df	*χ* ^*2*^	*P*	*R* ^*2*^	df	*F*	*P*
Elevation	1	15.62	**< 0.001**	1	2.37	0.123	–	1	1.92	**0.049**
Plant species	3	3.10	0.377	3	7.88	**0.049**	0.24	3	0.95	0.531
Elevation × Plant species	3	4.77	0.190	3	3.75	0.289	–	3	2.34	**0.006**
√read count	–	–	–	1	36.36	**< 0.001**	0.41	1	21.14	**0.001**
*Sampling location*	–	–	–	*1*	*0.99*	*0.319*	–	–	–	–

Read count was included as a covariate in the model on AM fungal richness and community composition to account for variation in sequencing depth. Sampling location was included as a random effect in the GLMM (in italic). Significant *P‐*values listed in bold.

The presence of AM fungi weakly increased with soil moisture, and was higher in thinner soils (i.e. low active soil layer depth) with low vegetation cover (Table 3). Soil moisture was associated with differences in AM fungal richness, whereas AM fungal community composition was mainly associated with variation in vegetation cover (Table 3).

**Table 3:** Impact of soil pH, soil moisture, depth of the active soil layer, distance to nearest snow patch and vegetation cover on arbuscular mycorrhizal (AM) fungal presence–absence, richness in samples positive for AM fungi, and community composition in the four focal plant species (*Bistorta vivipara, Dryas octopetala* × *integrifolia, Saxifraga oppositifolia, Silene acaulis*), as based on generalized linear mixed effects models (GLMM) and redundancy analysis (RDA).

	Presence–absence			Richness				Community composition		
	df	χ^2^	*P*	df	χ^2^	*P*	*R* ^2^	df	*F*	*P*
pH	1	0.11	0.736	1	0.02	0.902	–	1	0.72	0.661
Soil moisture	1	7.76	**0.005**	1	6.63	**0.010**	0.24	1	1.72	0.094
Depth of active soil layer	1	5.31	**0.021**	1	0.67	0.412	–	1	1.08	0.339
Distance to nearest snow patch	1	0.00	0.977	1	1.74	0.187	–	1	0.62	0.734
Vegetation cover	1	11.33	**0.001**	1	2.44	0.118	–	1	2.24	**0.027**
√read count	–	–	–	1	34.15	**< 0.001**	0.45	1	22.26	**0.001**
*Sampling location*	–	–	–	*1*	*0.00*	*1.000*	*–*	*–*	*–*	*–*
*Plant species*	–	–	–	*1*	*1.96*	*0.161*	*–*	3	0.96	0.486

Read count was included as a covariate in the model on AM fungal richness and community composition to account for variation in sequencing depth. Sampling location and plant species were included as random effects in the GLMM (in italic), whereas plant species was added as a fixed effect (covariate) for the model on community composition. Significant *P‐*values listed in bold.

## Discussion

This study provides a first glimpse of the incidence and diversity of AM fungi in the High Arctic. Although only 25% of our root samples contained AM fungi, the AM fungal community of our study site proved relatively diverse. Of only 384 VT of AM fungi known globally (in the Maarj*AM* database as of May 2020; Öpik *et al*., 2014), our target community included 23 VT (i.e. 6% of all known taxa), as well as six VT previously unrecorded in the Maarj*AM* database, from seven different genera. Arbuscular mycorrhizal fungal occurrence increased with elevation, species richness differed among plant species, and community composition was jointly influenced by elevation and plant species.

Our study provides an important step forward for understanding AM fungal diversity in Arctic regions. Thus far, Arctic AM fungal diversity has remained largely unexplored, as most studies conducted in these areas have focused on within‐root AM fungal structures (e.g. Newsham *et al*., 2009), and not on AM fungal diversity *per se*. Similar to our work, studies relying on morphological identification of AM fungal spores also found several AM fungal genera at high latitudes. For example, Greipsson *et al*. (2002) studied the spore community of AM fungi in Iceland and found spores of the genera *Glomus*, *Scutellospora*, *Acaulospora* and *Entrophospora*. The number of VT found here matches well to the 22 VT found by Öpik *et al*. (2013) in Finnish Lapland. These consisted mainly of VT from Glomeraceae, as well as two VT from Acaulosporaceae, whereas we found VT within the genera Glomeraceae, Claroideoglomeraceae, Diversisporaceae, Paraglomeraceae, Acaulosporaceae, Archaeosporaceae and Pacisporaceae.

As the current study included roots from several non‐AM plants, the richness of AM fungi observed may be biased downwards as compared to an equal‐sized sample targeting known AM plants. At the same time, the number of new VT found in our study was relatively high: globally, Davison *et al*. (2015) found 10 new VT out of 236 VT, and we found six of 29. Four of the most common VT detected in the current study also were detected by Liu *et al*. (2011) in the Tibetan Plateau. Because these AM fungal taxa have been found in a range of climatic habitats, such as temperate, subtropical and tropical zones (Öpik *et al*., 2010; Liu *et al*., 2011), several of the VT now detected in the High Arctic appear to be generalist, cosmopolitan species. Yet, the high number of new VT that we found does not support the idea that all Arctic AM fungal taxa would represent a subset of the taxa thriving in a broad range of habitats, including the harsh Arctic environment. In fact, five of the six new VT were detected only at the highest elevational range. Therefore, it appears that the VT found in the present study consist of a mix of generalist, cosmopolitan species as well as novel, specialist species potentially endemic to the Arctic. Just how wide their range actually is can be established only by further studies across habitats and regions.

As a technical caveat, previous studies of AM fungal species richness along latitudes and elevations frequently have confounded AM fungal turnover with concurrent changes in plant diversity (which generally increases from the poles to the tropics, and from the valley to the top of the mountain) and plant community composition. To avoid this, we focused on species present along broad gradients. A similar approach also was adopted by, for example, Liu *et al*. (2011) and Kotilínek *et al*. (2017). We also found low overall occurrence of AM fungi, which suggests an overall low rate of colonization of plant individuals. Furthermore, one may expect low diversity of AM fungi, also as a consequence of the low species richness of plant groups traditionally associated with AM fungi in the High Arctic (Bledsoe *et al*., 1990; Kohn & Stasovski, 1990; Väre *et al*., 1992; Dalpé & Aiken, 1998). Nonetheless, our study suggests that the Arctic does harbour a rich and underexplored diversity of AM fungi, with potentially specific adaptations to an extreme environment.

As one of our main findings, we identified elevation as a key determinant of whether *any* AM fungi were present, with higher frequencies of AM fungi at higher elevations. This is interesting, as one could expect lower AM fungal occurrence as vegetation cover decreases with elevation. Several other studies (e.g. Gai *et al*., 2012; Li *et al*., 2014; Coutinho *et al*., 2015) have found an effect of elevation on AM fungi, although the pattern itself has varied. These previous studies were, however, conducted in regions other than the Arctic, rendering direct comparisons difficult. Coutinho *et al*. (2015) found that spore density and AM fungal richness were highest at intermediate elevations in a Brazilian grassland system ranging in elevation from 800 to 1400 m asl. In working across the current elevational gradient of 0–500 m asl herein, we found the highest occurrence of AM fungi at high elevations, with no significant differences in richness across elevations.

Taken at face value, higher frequencies of AM fungi at higher elevations might suggest a higher need for beneficial AM fungal symbioses in a more stressful environment (Menge *et al*., 1978; Abrego *et al*., 2020a,b). Yet, we stress that many AM fungal–plant associations may not be symbiotic at all (Cosme *et al*., 2018; Wang *et al*., 2022), and that further studies thereby are needed to determine the actual nature of the relationship. By comparison, the species richness of AM fungi differed among plant species, whereas the community composition of AM fungi proved to be jointly shaped by plant species and elevation. Future studies should thus be aimed at further exploration of these combined effects.

Differences in the elevational patterns reported in previous studies (e.g. Gai *et al*., 2012; Li *et al*., 2014; Coutinho *et al*., 2015) probably is caused by variation of abiotic and biotic changes along elevational gradients. Many of the abiotic and biotic factors which change along elevational gradients (temperature, soil nutrient availability and vegetation) also will shape AM fungal communities across scales from local to global (Davison *et al*., 2015; Vályi *et al*., 2016). Globally, AM fungal diversity is structured by climatic and edaphic factors (Davison *et al*., 2021), whereas at the local scale, factors such as plant community composition play an important role (Rasmussen *et al*., 2018; Sepp *et al*., 2019). Here, the resolution of causal relationships remains a challenge. Factors such as soil pH, phosphorus, nitrogen and plant communities all may change along elevational and environmental gradients, and can impact AM fungal community composition (De Beenhouwer *et al*., 2015; Vályi *et al*., 2015; Vasar *et al*., 2022). At the same time, temperature, nutrients and soil moisture oftentimes may show no directional change with elevation (Körner, 2007). In our study, we sampled only a subset of abiotic factors of potential importance for AM fungi. Further studies therefore would be needed to validate the current suggestion that abiotic impacts on AM fungi in the Arctic may be weak. By comparison, our finding of an imprint of plant species identity on AM fungal richness matches well with previous studies (Eom *et al*., 2000; Hausmann & Hawkes, 2009; Lekberg *et al*., 2013), and highlights plant species identity as a factor important in determining AM fungal richness in the High Arctic.

Importantly, AM fungal DNA was detected in seemingly non‐AM plants such as *Cerastium alpinum, Pedicularis hirsuta, Polemonium boreale, Saxifraga nivalis* and *Silene involucrata*. Although these records could, in principle, derive from soil residues on roots, we did clean the roots to the best of our abilities (see Materials and Methods). Other studies have shown hyphal colonization of non‐AM plants with no associated benefit to the plants (Cosme *et al*., 2018; Wang *et al*., 2021, 2022), and potentially this could be the case here, too. Nevertheless, the occurrences detected highlight the importance of further exploring the diversity of AM fungi in plant species otherwise considered non‐AM. In addition, methods aimed at further disentangling the intimacy and morphology of the AM fungal–plant host relationship may provide new insights into the functional relationships behind the plant–AM fungal associations observed. In the current case, we lacked access to root material for microscopy, but will return to explore the specific level of colonization by AM fungi of Arctic plant roots in future work. Such future initiatives also will include a comparison of detection rates between methods based on DNA vs microscopy. As a further avenue, we identify the need for studies aimed at identifying the relative importance of different factors shaping AM fungal communities in the Arctic, and comparisons between the importance of plant species identity as opposed to the abiotic environment (cf. Abrego *et al*., 2020a,b, for other fungal groups). Beyond effects of plant species, there may be further effects of intraspecific variation among individuals of the same plant species – although in a mesocosm experiment, Rasmussen *et al*. (2019) found no effect of intraspecific genetic variation in plants on the colonization level of AM fungi.

The patterns detected for AM fungi are qualitatively dissimilar to those of ectomycorrhizal and endophytic fungi studied in the same samples (Abrego *et al*., 2020a). In the study, Abrego *et al*. (2020a) identified elevation – and the associated abiotic environment – as a driver stronger than the plant species in determining the richness and composition of ectomycorrhizal and endophytic root‐associated fungal communities. This may suggest that different drivers structure different groups of root‐associated fungi in the High Arctic. Again, we need more work to validate this suggestion. Further studies into the different root‐associated fungi may reveal other interesting findings, such as whether different fungal groups influence each other leading to negative co‐occurrences, and whether such interactions could underlie the influence of elevation and plant identity on fungal community structure.

## Conclusion

This study provides one of the first fundamental insights into the diversity, composition and drivers of AM fungi within a plant–AM fungal network of the High Arctic. It points to unexpected diversity in a poorly known group of organisms of global importance. In addition, our research identifies elevation as a main determinant of the occurrence of AM fungi, and both elevation and plant communities as key forces driving local AM fungal community structure. Given the relatively small area targeted by our study, and the relatively high diversity of AM fungi discovered, our results suggest that there is still more diversity to be discovered in Arctic regions. Although our study did not target the functional role of AM fungi, the high diversity of AM fungi detected calls for urgent examination of the functional role of AM fungi within the High Arctic.

## Author contributions

PUR, NA, TR, and AJMT conceived and designed the experiment; NA and TH conducted the empirical work; PUR and TH conducted the molecular work; PUR, LWH, MÖ and S‐KS performed the bioinformatic analyses; and PUR and FGB analyzed the data; PUR wrote the first draft, and all authors contributed to the final manuscript.

## Supporting information


**Fig. S1** Schematic representation of the Zackenberg sampling area in Greenland.
**Fig. S2** The impact of elevation on environmental factors measured at each sampling location.
**Fig. S3** Environmental factors at each sampling location.
**Fig. S4** Virtual taxa accumulation curves for each plant species.
**Fig. S5** Diagnostic plots of statistical models.
**Fig. S6** Heat map illustrating associations among arbuscular mycorrhizal fungi and the four best‐sampled plant species at low, mid‐ and high elevation in Zackenberg, Greenland.
**Methods S1** Description of how putative new VT were identified to Glomeromycotina.

**Notes S1** Phylogenetic tree in Newick format.
**Table S1** List of plant species collected for each sampling location.
**Table S2** Sampling and DNA sequencing of plant species.
Please note: Wiley Blackwell are not responsible for the content or functionality of any Supporting Information supplied by the authors. Any queries (other than missing material) should be directed to the *New Phytologist* Central Office.

## Data Availability

Sequencing data for each sample, representative sequences for each VT, and associated biotic and abiotic data have been deposited as a BioProject at INSDC under accession no. PRJEB40490.
