# Supplementary material for: Elevation and plant species identity jointly shape a diverse arbuscular mycorrhizal fungal community in the High Arctic
Source: New Phytol. 2022 Jul 15;236(2):671–83. doi: 10.1111/nph.18342 (PMC9796444; doi:10.1111/nph.18342)
Supplement: Supplementary file 1 — Fig. S1 Schematic representation of the Zackenberg sampling area in Greenland. Fig. S2 The impact of elevation on environmental factors measured at each sampling location. Fig. S3 Environmental factors at each sampling location. Fig. S4 Virtual taxa accumulation curves for each plant species. Fig. S5 Diagnostic plots of statistical models. Fig. S6 Heat map illustrating associations among arbuscular mycorrhizal fungi and the four best‐sampled plant species at low, mid‐ and high elevation in Zackenberg, Greenland. Methods S1 Description of how putative new VT were identified to Glomeromycotina. [file NPH-236-671-s002.pdf]

New Phytologist **Supporting Information**

Article title: Elevation and plant species jointly shape a diverse arbuscular mycorrhizal fungal community in the High Arctic
Authors: Pil U. Rasmussen, Nerea Abrego, Tomas Roslin, Maarja Öpik, Siim-Kaarel Sepp, F. Guillaume Blanchet, Tea Huotari, Anders F. Andersson, Luisa W. Hugerth and Ayco J. M. Tack
Article acceptance date: 19th of June 2022.

The following Supporting Information is available for this article:

Fig. S1 Schematic representation of the Zackenberg sampling area in Greenland.

Fig. S2 The impact of elevation on environmental factors measured at each sampling location.

Fig. S3 Environmental factors at each sampling location.

Fig. S4 Virtual taxa (VT) accumulation curves for each plant species.

Fig. S5 Diagnostic plots of statistical models.

Fig. S6 Heat map illustrating associations among arbuscular mycorrhizal fungi and the four best-sampled plant species at low, mid, and high elevation in Zackenberg, Greenland.

Table S1 List of plant species collected for each sampling location.

Table S2 Sampling and DNA sequencing of plant species.

Methods S1 Description of how potential new virtual taxa (VT) were identified to Glomeromycotina.

Notes S1 Phylogenetic tree in Newick format.

Fig. S1 Schematic representation of the Zackenberg sampling area in Greenland. Sampling locations (18 in total) from different elevations are marked as black lines.

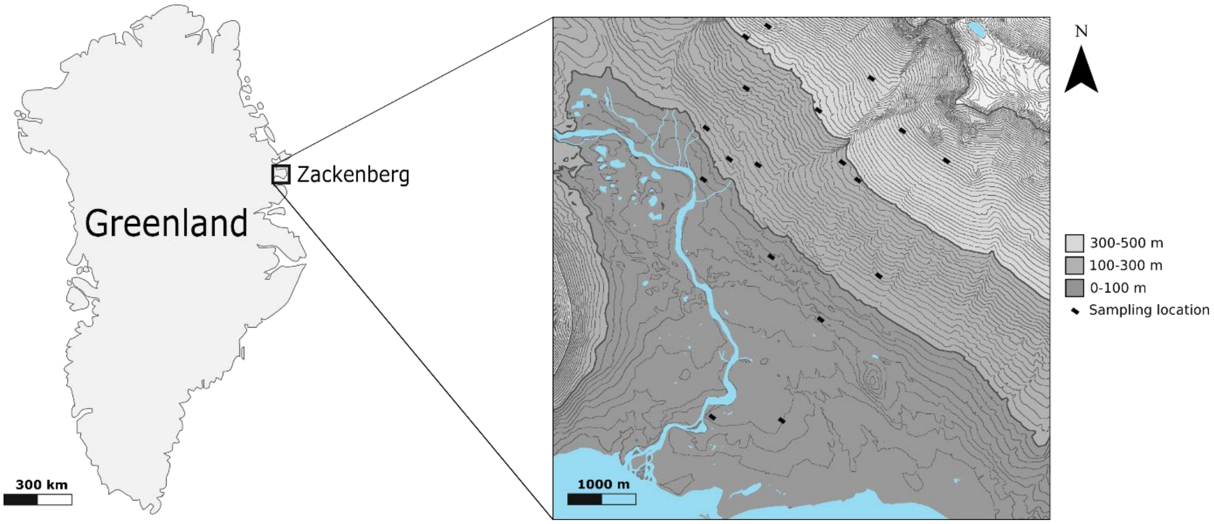

Fig. S2 The relationship between elevation and a) pH, b) soil moisture, c) depth until the active soil layer, d) distance to nearest snow patch, and e) vegetation cover measured at each sampling location (n = 18). The relationship between environmental factors and elevation is shown by a trendline (black) with its standard error interval (grey) generated by function *geom_smooth* from package *ggplot2* in R.

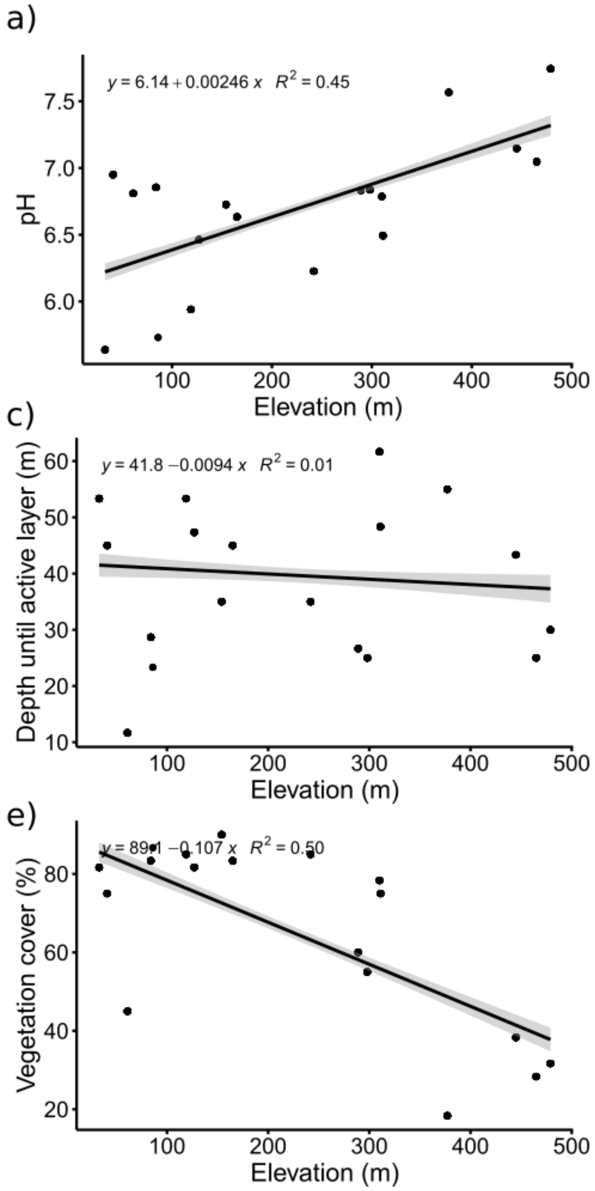

Fig. S3 Environmental factors (a) pH, b) soil moisture, c) depth until the active soil layer, d) distance to nearest snow patch, and e) vegetation cover) at each sampling location (n = 18).

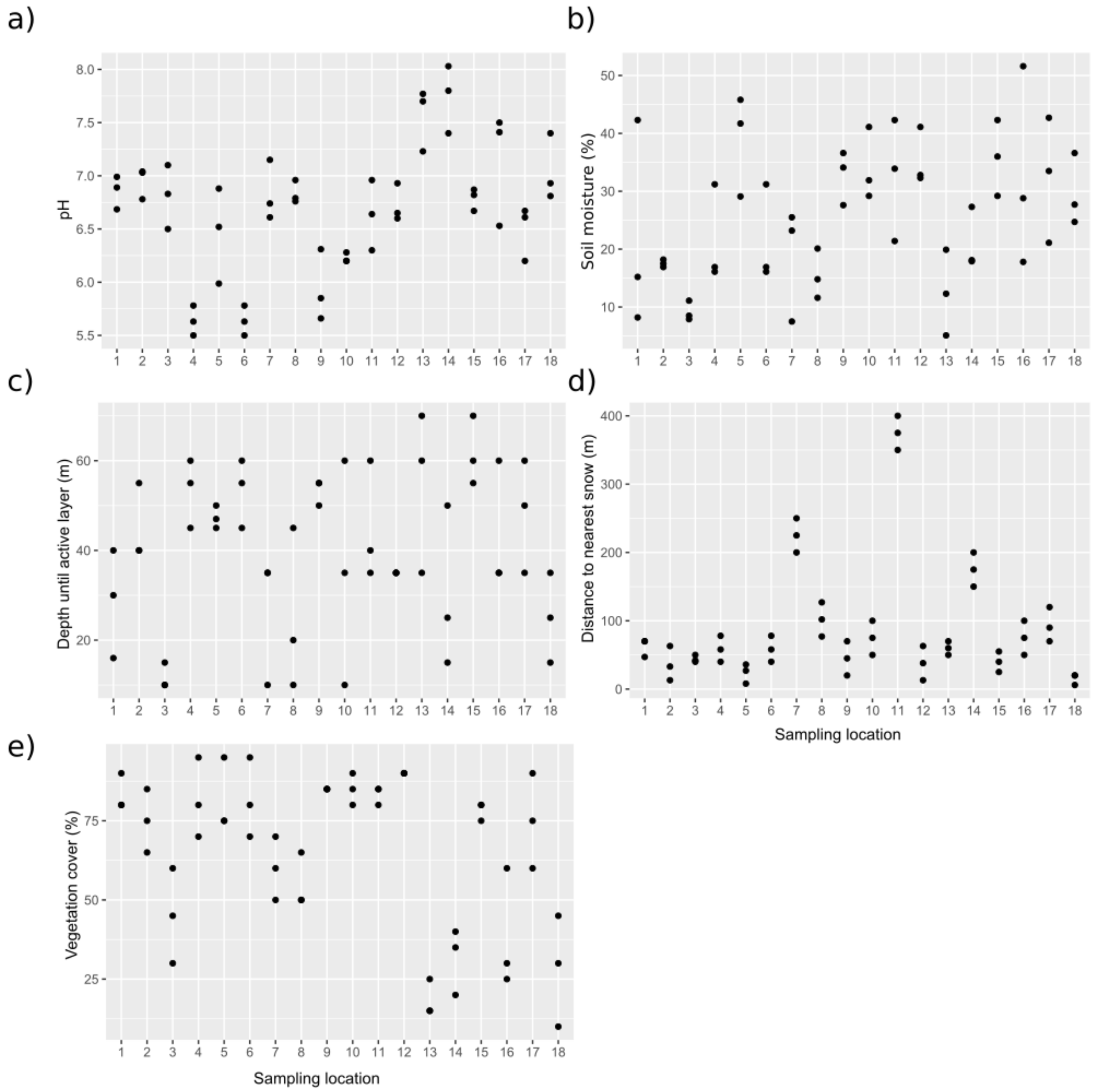

Fig. S4 Virtual taxa (VT) accumulation curves for each plant species. Arnang = *Arnica angustifolia*, Bisviv = *Bistorta vivipara*, Calpur = *Calamagrostis purpurascens*, Ceralp = *Cerastium alpinum*, Dryoct = *Dryas octopetala x integrifolia*, Paprad = *Papaver radicatum*, Pedhir = *Pedicularis hirsuta*, Polbor = *Polemonium boreale*, Saxniv = *Saxifraga nivalis*, Saxopp = *Saxifraga oppositifolia*, Silaca = *Silene acaulis*, Silinv = *Silene involucrata*. Due to the low number of samples, no accumulation curves could be made for *Arenaria humifusa* and *Oxyria digyna.*

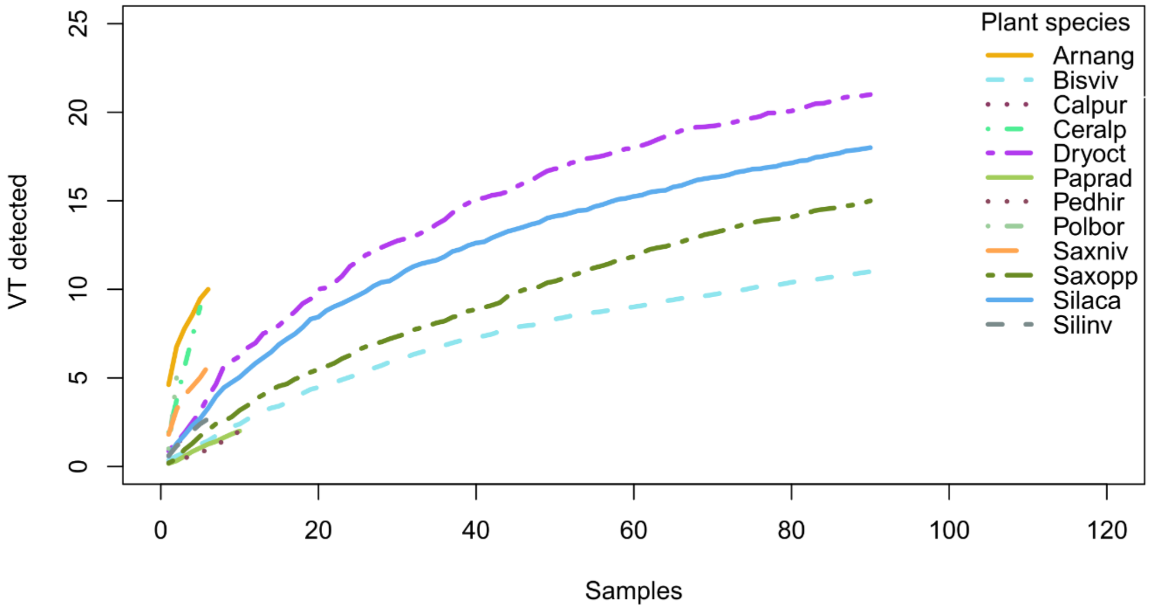

Fig. S5 Diagnostic plots of statistical models. a) and b) quantile-quantile and fitted vs. residual plots for the model of the impact of elevation and plant species identity on AM fungal richness, and c) and d) for the model of the impact of abiotic measures on AM fungal richness.

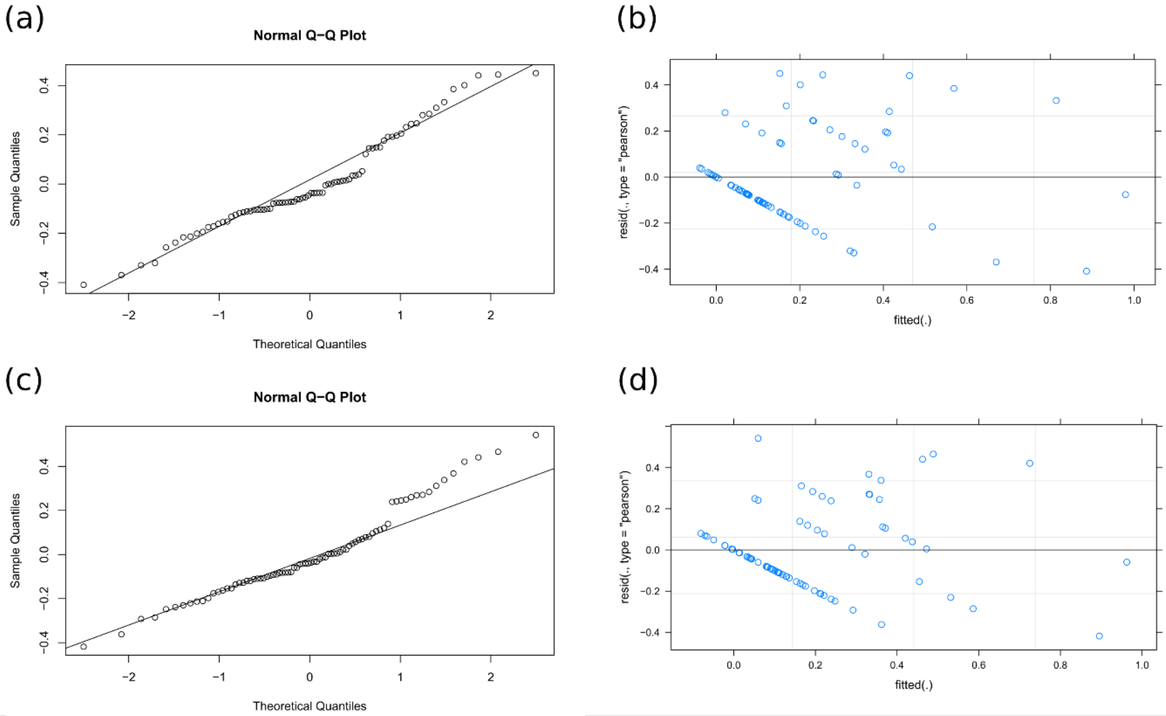

Fig. S6 Heat map illustrating associations among the four best-sampled plant species ('focal plant species') and arbuscular mycorrhizal (AM) fungal virtual taxa (VT) found at low (0-100 m), mid (100-300 m) and high (300-500 m) elevation in Zackenberg, Greenland. Intensity of colour (greyscale) within boxes corresponds to the frequency at which a given AM fungal taxon was found in samples from each combination of plant species and elevation, with white showing that the AM fungal taxon was not found. AM fungal taxa are coloured and ordered by relatedness at family level, and ordered numerically within family. Bisviv = *Bistorta vivipara*, Dryoct = *Dryas octopetala x integrifolia,* Saxopp = *Saxifraga oppositifolia*, Silaca = *Silene acaulis*.

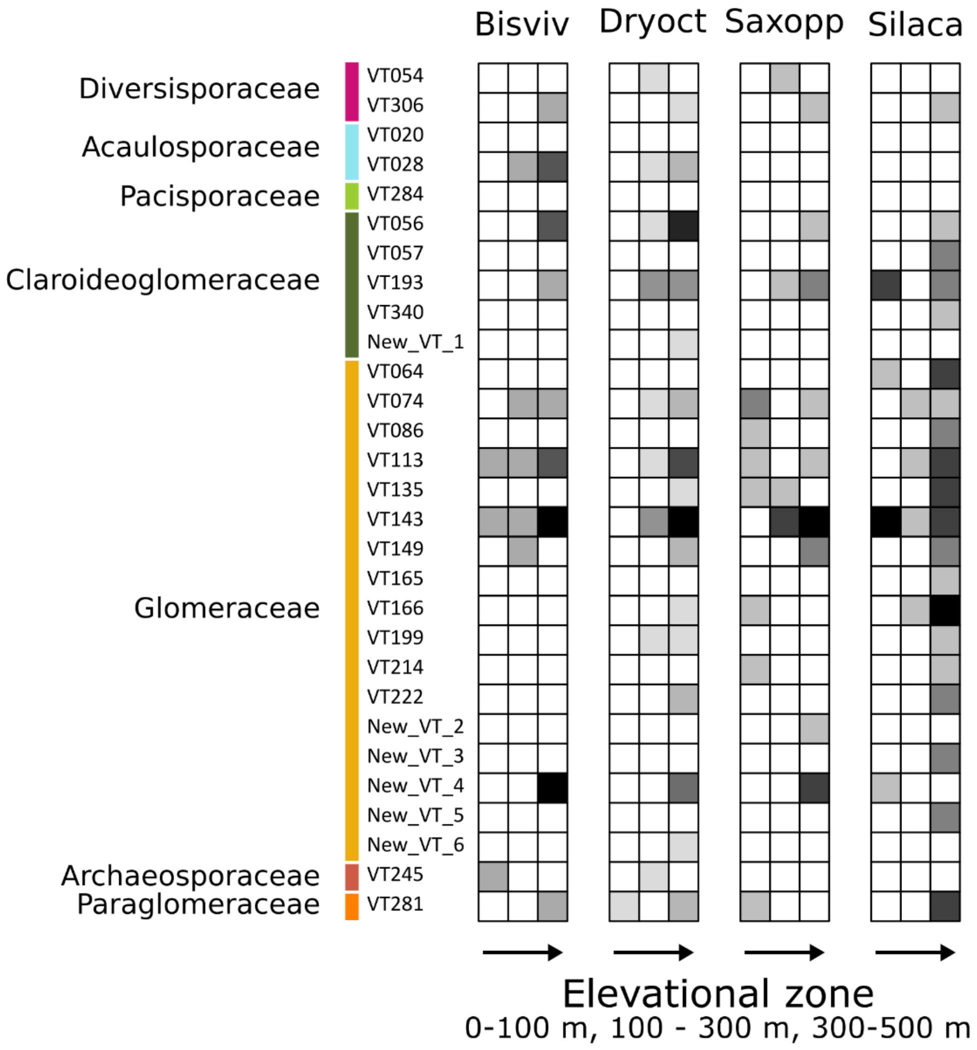

Table S1 List of plant species collected for each sampling location. Alobor = *Alopecurus borealis*, Arclat = *Arctagrostis latifolia*, Arehum = *Arenaria humifusa*, Armscr = *Armeria scabra*, Arnang = *Arnica angustifolia*, Bisviv = *Bistorta vivipara*, Calpur = *Calamagrostis purpurascens*, Carsp = *Carex* sp, Castet = *Cassiope tetragona*, Ceralp = *Cerastium alpinum*, Chalat = *Chamerion latifolium*, Draalp = *Draba alpina*, Draarc = *Draba arctica*, Dryoct = *Dryas octopetala x integrifolia*, Fessp = *Festuca* sp, Oxydig = *Oxyria digyna*, Paprad = *Papaver radicatum*, Pedfla = *Pedicularis flammea*, Pedhir = *Pedicularis hirsuta*, Polbor = *Polemonium boreale*, Potsp = *Potentilla* sp, Rholap = *Rhododendron lapponicum*, Salarc = *Salix arctica*, Saxniv = *Saxifraga nivalis*, Saxopp = *Saxifraga oppositifolia*, Silaca = *Silene acaulis*, Silinv = *Silene involucrata*, Vaculi = *Vaccinium uliginosum*.

	Sampling location																		Total
	1	2	3	4	5	6	7	8	9	10	11	12	13	14	15	16	17	18	
	Elevation (m)																		
	84	41	61	33	127	33	289	298	119	242	165	154	377	479	310	445	311	465	
Alobor					1				1			1			1	1			**5**
Arclat					1		1		1			1			1				**5**
Arehum																	1		**1**
Armscr						1	1												**2**
Arnang								1						1	1	1	1	1	**6**
Bisviv	5	5	5	1	5	9	5	5	5	5	5	5	5	5	5	5	5	5	**90**
Calpur	1		1			1				1								1	**5**
Carsp1							1												**1**
Carsp2											1								**1**
Carsp3						1													**1**
Castet	5	5	5	1	5	9	5	5	5	5	5	5			5		5		**70**
Ceralp			1		1			1						1	1				**5**
Chalat		1																	**1**
Dryoct	5	5	5	1	5	9	5	5	5	5	5	5	5	5	5	5	5	5	**90**
Draalp												1							**1**
Draarc	1	1									1		1						**4**
Fessp1		1														1			**2**
Oxydig													1						**1**
Paprad	1		1			1	1		1	1	1			1	1			1	**10**
Pedfla						1										1			**2**
Pedhir					1	1	1	1	1		1	1		1		1		1	**10**
Polbor													1	1					**2**
Potsp1						1											1		**2**
Potsp2													1						**1**
Rholap						1				1									**2**
Salarc	5	5	5	1	5	9	5	5	5	5	5	5	5	5	5	5	4	5	**89**
Saxniv	1	1	1			1				1							1	1	**7**
Saxopp	5	5	5		5	10	5	5	5	5	5	5	5	5	5	5	5	5	**90**
Silaca	5	5	5		5	10	5	5	5	5	5	5	5	5	5	5	5	5	**90**
Silinv	1	1	1					1		1			1				1		**7**
Vaculi					1	1		1	1		1	1							**6**
**Total**	**35**	**35**	**35**	**4**	**35**	**66**	**35**	**35**	**35**	**35**	**35**	**35**	**30**	**30**	**35**	**30**	**34**	**30**	**609**

Table S2 Sampling and DNA sequencing of plant species. For a full description of the sampling strategy, see *Study design* in the *Materials and Methods* section. References used for the table includes Bledsoe *et al.*, 1990; Clemmensen & Hansen, 1998; Olsson *et al.*, 2004; Bueno *et al.*, 2017.

				Part 1: Pilot study			Part 2: Sequencing		
Plant species	Mycorrhizal status based on previous literature	Number of sampling locations found	Total number of samples	Included in pilot	Number of samples in pilot (total n = 162)	Indication of AMF in pilot	Included in sequencing	Number of samples in sequencing (n = 424)	Percentage of samples with AMF
Alopecurus borealis	No info	5	5	Yes	5	No	No	0	-
Arctagrostis latifolia	AM	5	5	Yes	5	No	No	0	-
Arenaria humifusa	No info	1	1	Yes	1	Yes	Yes	1	100
Armeria scabra	No info (though *A. maritima* is AM+NM)	2	2	Yes	2	Yes	Yes	2	0
Arnica angustifolia	AM	6	6	Yes	6	Yes	Yes	6	100
Bistorta vivipara	AM+ECM+NM	18	90	Yes	18	Yes	Yes	90	20
Calamagrostis purpurascens	No info (though *C. Epigejos* is AM+NM)	5	5	Yes	5	Maybe	Yes	5	40
Cerastium alpinum	NM	5	5	Yes	5	Yes	Yes	5	60
Chamerion latifolium	AM+NM	1	1	Yes	1	Yes	Yes	1	0
Draba alpina	No info (though other Draba species are NM)	1	1	Yes	1	Yes	Yes	1	0
Draba arctica	No info (though other Draba species are NM)	4	4	Yes	4	Yes	Yes	4	0
Dryas octopetala x *integrifolia*	AM+ECM	18	90	Yes	18	Yes	Yes	90	21
Oxyria digyna	AM+NM	1	1	Yes	1	Yes	Yes	1	100
Papaver radicatum	No info (but *Papaver rhoeas* is AM+NM)	10	10	Yes	10	Yes	Yes	10	20
Pedicularis flammea	No info (typically ignored since it is hemiparasitic)	2	2	Yes	2	Yes	Yes	2	0
Pedicularis hirsuta	NM	10	10	Yes	10	Yes	Yes	10	10
Polemonium boreale	NM	2	2	Yes	2	Yes	Yes	2	100
Salix arctica	ECM	18	89	Yes	18	No	No	0	-
Saxifraga nivalis	NM	7	7	Yes	7	Yes	Yes	7	57
Saxifraga oppositifolia	AM+NM	17	90	Yes	17	Yes	Yes	90	21
Silene acaulis	AM+ECM+NM	17	90	Yes	17	Yes	Yes	90	27
Silene involucrata	NM	7	7	Yes	7	Yes	Yes	7	43
Cassiope tetragona	ERM	14	70	No	0	-	No	0	-
Rhododendron lapponicum	ERM+NM	2	2	No	0	-	No	0	-
Vaccinium uliginosum	ERM	6	6	No	0	-	No	0	-

AM = arbuscular mycorrhizal, ECM = ectomycorrhizal, ERM = ericoid mycorrhiza, NM = non-mycorrhiza.

References:

Bueno, CG, Moora, M, Gerz, M, et al. Plant mycorrhizal status, but not type, shifts with latitude and elevation in Europe. Global Ecol Biogeogr. 2017; 26: 690– 699. https://doi-org.ezproxy.utlib.ut.ee/10.1111/geb.12582

Olsson, PA, Eriksen, B, and Dahlberg, A. Colonization by arbuscular mycorrhizal and fine endophytic fungi in herbaceous vegetation in the Canadian High Arctic. Canadian Journal of Botany. 82(11): 1547-1556. https://doi.org/10.1139/b04-111

Michelsen, A., Schmidt, I.K., Jonasson, S. et al. Leaf 15N abundance of subarctic plants provides field evidence that ericoid, ectomycorrhizal and non-and arbuscular mycorrhizal species access different sources of soil nitrogen. Oecologia 105, 53–63 (1996). https://doi.org/10.1007/BF00328791.

For NM plant specifically:

Armeria scabra: Clemmensen, K. E., & Hansen, A. H. (1998). Mykorrhizasymbioser i fire gronlandske plantesamfund i relation tU forskellige jordbundsfaktorer. Arktisk Biologisk Feltkursus, Qeqertarsuaq. University of Copenhagen, Copenhagen.

Papaver radicatum: Bledsoe, C., Klein, P., & Bliss, L. C. (1990). A survey of mycorrhizal plants on Truelove Lowland, Devon Island, NWT, Canada. Canadian Journal of Botany, 68(9), 1848-1856.

Pedicularis flammea: Clemmensen, K. E., & Hansen, A. H. (1998). Mykorrhizasymbioser i fire gronlandske plantesamfund i relation tU forskellige jordbundsfaktorer. Arktisk Biologisk Feltkursus, Qeqertarsuaq. University of Copenhagen, Copenhagen.

Methods S1 Description of how putative new virtual taxa (VT) were identified to Glomeromycotina.

The potential new Glomeromycotina virtual taxa (VT) were aligned against the in-house database type sequences and 5 outgroup sequences (HQ202311_Ceratocystiopsis_brevicomis, EU940055_Epiglia_gloeocapsae, AY544694_Geoglossum_nigritum, GQ280405_Xylogone_sphaerospora, AM495013_Teloschistes_hosseusianus). The alignment was performed in MAFFT v7 (Katoh et al., 2002) using the default settings. A neighbour-joining tree with 100 bootstraps (Notes S1 Phylogenetic tree in Newick format.tre) was then constructed in TOPALi v2 (http://www.topali.org/) to assess whether the potential new VT (denoted in the tree with the prefix "Uniq") cluster phylogenetically with existing taxa or fall outside the Glomeromycotina. In the former case, the VT was manually checked (using BLAST+) against the entire in-house database (containing 22961 sequences from the MaarjAM database and the new VT already found from previous yet unpublished data) to check whether the new VT was sufficiently different from existing taxa in the database. If the potential new VT was sufficiently different (less than 97% similarity) from the in-house database sequences, and at least 90% of the sequence was aligned, the ASV was marked as a new VT and appended to the in-house database. The whole sequence dataset was then blasted against the appended database to obtain the final AM fungal sequences and taxa.

Notes S1 Phylogenetic tree in Newick (.tre) format. The tree was constructed in TOPALi v2 (http://www.topali.org/) as described in Methods S1.
